# Valorisation of *Olea europaea* L. Olive Leaves through the Evaluation of Their Extracts: Antioxidant and Antimicrobial Activity

**DOI:** 10.3390/foods10050966

**Published:** 2021-04-28

**Authors:** Mónica Sánchez-Gutiérrez, Isabel Bascón-Villegas, Alejandro Rodríguez, Fernando Pérez-Rodríguez, África Fernández-Prior, Antonio Rosal, Elena Carrasco

**Affiliations:** 1 Food Science and Technology Department, Universidad de Córdoba, Darwin Building, 14014 Córdoba, Spain; q12bavii@uco.es (I.B.-V.); b42perof@uco.es (F.P.-R.); bt2cajie@uco.es (E.C.); 2 BioPrEn Group, Chemical Engineering Department, Universidad de Córdoba, Marie-Curie Building, 14014 Córdoba, Spain; a.rodriguez@uco.es; 3 Instituto de la Grasa, Consejo Superior de Investigaciones Científicas (CSIC), Campus Universitario Pablo de Olavide, Edificio 46, Ctra. de Utrera, km. 1, 41013 Seville, Spain; mafprior@ig.csic.es; 4 Molecular Biology and Biochemical Engineering Department, Campus Universitario Pablo de Olavide, Edificio 46, Ctra. de Utrera, km. 1, 41013 Seville, Spain; arosray@upo.es

**Keywords:** agri-food waste, bioactive compounds, polyphenols, foodborne pathogens, microwave assisted extraction, nutraceutical

## Abstract

*Olea europaea* L. leaves constitute a source of bioactive compounds with recognized benefits for both human health and technological purposes. In the present work, different extracts from olive leaves were obtained by the application of two extraction methods, Soxhlet and microwave-assisted extraction (MAE), and six solvents (distilled water, ethanolic and glycerol mixtures solvents). MAE was applied under 40, 60 and 80 °C for 3, 6.5 and 10 min. The effect of the extraction method, solvent and treatment factors (the latter in MAE) on the total phenol content (TPC), the antioxidant activity (AA) and the phenolic profile of the extracts were all evaluated. The extracts showed high values of TPC (up to 76.1 mg GAE/g DW) and AA (up to 78 mg TE/g DW), with oleuropein being the most predominant compound in all extracts. The Soxhlet extraction method exhibited better yields in TPC than in MAE, although both methods presented comparable AA values. The water MAE extract presented the strongest antimicrobial activity against five foodborne pathogens, with minimum inhibitory concentration (MIC) values ranging from 2.5 to 60 mg/mL. MAE water extract is proposed to be exploited in the food and nutraceutical industry in the frame of a sustainable economy.

## 1. Introduction

Spain is the world’s leading producer of olive oil (approximately 60% of EU and 45% of world production), with an annual average of 1,374,980 t over the last six seasons (2015/16–2019/20). Similarly, Spain is the world’s leading exporter of olive oil. Olive oil exports amounted to 1,109,900 t in the last campaign (2019), with more than 100 destination countries [1]. The cultivation area dedicated to olive groves in Andalusia represents 60% of the Spanish area, covering 2,584,564 ha. In the 2019/2020 season, Andalusia contributed with 80% of the Spanish production and with 75% of the Spanish exports, with a turnover of around 3549 million euros, and a generation of 16.4 million days of work approximately [2].

When olives are received at the olive mill, they are subjected to a pre-cleaning process in which a considerable amount of olive leaves are separated, corresponding to 8% *w*/*w* roughly of the milled olives. In Andalusia, 416,000 t of olive leaves were collected in the last season, being primarily destined to animal feed despite their bitter taste, which, in general, limits their use [3]. Due to the large amount of leaf waste generated in the production of olive oil, a system embracing a recycling approach is proposed, based on a circular bioeconomy model, in which the return of waste and by-products into the production cycle reduces the generation of residues [4]. Under this system, the management and recovery of this waste is carried out with the aim of both producing new materials and recovering active substances. This system is covered by the so-called bioeconomy concept, that “means using renewable biological resources from land and sea, like crops, forests, fish, animals and micro-organisms to produce food, materials and energy” [5]. In this sense, the bioeconomy replaces the linear economic model based on taking, making and discarding, which has a great environmental impact, due to the limited resources available in nature [6].

From an economic point of view, the obtention of high-added-value products through the integral use of agri-food by-products is highly advantageous, considering the availability and low price of these by-products [7]. In this line, emerging green extraction technologies and methods are being developed to contribute to their sustainable valorization. These innovative methods are characterized by low energy consumption and reduced extraction time and solvent use [8]. Among them, microwave-assisted extraction (MAE) is one of the most promising techniques in which the extraction process is considerably shorter as a result of power control [9]. Organic solvents have traditionally been used in the recovery of bioactive compounds, most of which present high volatility and toxicity, thus jeopardizing the environment and health status [10]. These characteristics, combined with the additional cost of removing the solvent after extraction, limit their application. Therefore, these solvents, not compatible with the concept of eco-friendly extraction, make it necessary to seek green alternatives [11] such as water, which can be used as a universal solvent because it is non-toxic, non-flammable, environmentally friendly, abundant and cheap; in addition, due to its chemical properties, it is an excellent solvent for the extraction of polar compounds [12]. In the same way, glycerol has been proposed as a green alternative, with increasing interest lately since it is naturally present in food, is cheap and non-toxic, and when used in combination with water, can adjust its polarity and thus may increase the recovery of substances like polyphenols [13].

Polyphenolic compounds, the most abundant secondary metabolites in plants, are receiving great interest due to their well-known antimicrobial, antioxidant and anti-cancer activities. In recent years, phenolic compounds have been studied in relation to their antihypertensive and hypocholesterolemic effects, as well as their role in the prevention of diseases related to oxidative stress, such as inflammatory disorders, diabetes, Alzheimer’s, cancer and cardiovascular disease [14,15,16]. Olive leaf constitutes an excellent source of bioactive compounds such as oleuropein, verbascoside, rutin, tyrosol and hydroxytyrosol, at the same level as olives and olive oil. Scientific evidence attributing beneficial health properties to these bioactive compounds [17] accounts for the increasing interest shown by the pharmaceutical, cosmetic, nutraceutical and food industries in these compounds. In the latter sector, the antimicrobial and antioxidant activities of polyphenolic compounds are exploited through their potential use as natural additives [18].

In recent years, awareness has been raised among consumers about the importance of diet in health, not only appreciating the quality and quantity of nutrients in foods, but also their active positive effects on health [19]. In this respect, phenolic compounds could prevent chemical/enzymatic oxidative reactions as well as inhibit microbial proliferation of pathogens, thus conferring on them a potential role as natural food additives and supplements [20]. Furthermore, microbial inhibition could contribute to solving one of the major global public health risks, i.e., the emergence of multi-drug-resistant bacterial strains in the food chain that could cause foodborne microbial-resistant diseases [21]. Thus, the valorization of olive leaves, a massive agricultural by-product, is possible through their application in nutraceutical foods and supplements, as has been proposed by several authors in recent years, since plant leaves are currently the main waste product of the agricultural industry, and can become a major environmental problem and cause of pollution [22,23,24,25].

In this study, the bioactivity of phenolic compounds with nutraceutical potential from olive leaves, a by-product of olive oil production, was compared for the first time using MAE at short times and low temperatures, and environmentally friendly solvents such as glycerol, ethanol and water. The antioxidant activity (AA) as well as the total phenolic content (TPC) and phenolic profile of the extracts were determined. Similarly, the antimicrobial activity of the extracts was evaluated against different foodborne pathogenic microorganisms, i.e., *Listeria monocytogenes*, *Salmonella* Typhimurium, *Escherichia coli*, *Yersinia enterocolitica* and *Staphylococcus aureus*.

## 2. Materials and Methods

### 2.1. Chemicals and Reagents

Folin–Ciocalteu’s reagent, potassium persulfate (K_2_S_2_O_8_) and standards of gallic acid were purchased from Merck (Darmstadt, Germany). Anhydrous sodium carbonate (Na_2_CO_3_) was acquired from Panreac (Castellar del Vallès, Barcelona, Spain). ABTS diammonium salt (2,2-azino-bis (3-ethylbenzothiazoline-6-sulphonic acid)), acetic acid and standards of Trolox (6-hydroxy-2,5,7,8-tetramethylchroman-2-carboxylic acid) were obtained from Sigma-Aldrich (Madrid, Spain). Glycerol was acquired from Labbox (Barcelona, Spain), ultrapure water was obtained using a Milli-Q water system (Millipore, Milford, MA, USA) and ethanol was purchased from Romil Ltd. (Waterbeach, UK). High performance liquid chromatography (HPLC) grade acetonitrile was acquired from Honeywell Research Chemicals (Seelze, Germany), whereas the HPLC standards of hydroxytyrosol, protocatechuic acid, verbascoside, luteolin-7-O-glucoside, apigenin-7-O-glucoside, luteolin, oleuropein and apigenin were purchased from Sigma-Aldrich (Madrid, Spain).

### 2.2. Culture Media and Bacterial Strains

The microorganisms tested in this study were *Escherichia coli* (CECT 8295), *Listeria monocytogenes* (CECT 4032), *Salmonella* Typhimurium (CECT 704), *Staphylococcus aureus* (CECT 5193) and *Yersinia enterocolitica* (CECT 754). All microorganisms’ cultures were acquired from the Spanish Collection of Type Culture (Valencia, Spain).

Nutrient agar medium (NA) for *E. coli*, *Y. enterocolitica* and *S. aureus*, tryptone soy agar (TSA) for *S.* Typhimurium, brain heart infusion agar (BHIA) for *L. monocytogenes* and Mueller–Hinton broth (MHB) were acquired from Oxoid™ (Hampshire, UK), whereas cations supplements, magnesium chloride hexahydrate (MgCl_2_·6H_2_O) and calcium chloride dihydrate (CaCl_2_·2H_2_O) were obtained from Sigma-Aldrich (St. Louis, MA, USA).

### 2.3. Plant Material

Olive leaves from the “Hojiblanca” variety were kindly supplied by a local farmer from an olive grove in Cordoba (Spain) in mid-March 2019. The sampling areas were selected randomly just before pruning. Branches with leaves were collected from at least ten different trees. Prunings were immediately taken to the laboratory, where the leaves were removed from the branches. Leaves were hand-washed and left to dry in open air and darkness. They were then grounded and sieved so as to obtain particles with a diameter of <2 mm. Ground leaves were stored at ambient temperature (around 25 °C) in a dry, dark room until use.

### 2.4. Extraction of Bioactive Compounds from Olive Leaf Samples

Two methods, Soxhlet and microwave-assisted extraction, were employed to obtain extracts from the olive leaves.

#### 2.4.1. Soxhlet Extraction

The extraction was performed boiling a suspension prepared with 20 g of dried ground olive leaves in 160 mL of solvent for 5 h. Six extracts were obtained by using six different solvents: distilled water, 50% ethanol (*v*/*v*), 75% ethanol (*v*/*v*), 5% glycerol (*v*/*v*), 10% glycerol (*v*/*v*) and 15% glycerol (*v*/*v*). Once cooled, extracts were filtered through a Whatman No. 1 filter (Sigma-Aldrich, St. Louis, MA, USA). Afterwards, samples were filtered again with a 0.45 μm nylon syringe filter (Labbox, Barcelona, Spain) and kept in refrigeration until analysis.

#### 2.4.2. Microwave-Assisted Extraction (MAE)

The extraction was carried out on an ETHOS Microwave Extraction System (Milestone, Sorisole, Italy), at 800 W using magnetic stirring at a 90% level (2970 rpm), at three different temperatures (40, 60 and 80 °C) and times (3, 6 and 10 min). The extraction ratio was 1:8 (*w*/*v*), and the same solvents as for Soxhlet extraction were used. A full factorial design was applied. After the process, extracts were collected and treated as for Soxhlet extraction.

### 2.5. Total Phenolic Compounds (TPC)

The TPC of the extracts obtained was determined by the Folin–Ciocalteu method described by Singleton et al. [26] with modifications. In brief, sample aliquots of 0.25 mL were mixed with 1.25 mL of Folin–Ciocalteu reagent and 2.5 mL of 7.5% *w/v* sodium carbonate. After 30 min of incubation at 40 °C, absorbance was measured at 760 nm using a Perkin Elmer UV/VIS Lambda 25 spectrophotometer (Waltham, MA, USA). Gallic acid (GA) was the reference standard, and results were expressed as mg gallic acid equivalents (GAE)/g of dry weight (DW). All measurements were performed in triplicate.

### 2.6. In Vitro Antioxidant Assay (ABTS Radical Scavenging Method)

The ABTS scavenging activity assay of samples was determined as described by Espinosa et al. [27]. A radical solution was prepared (7 mM ABTS and 2.45 mM persulphate of potassium) and left in the dark overnight for 12–16 h before use. The radical solution was diluted with ethanol to an absorbance of 0.70 ± 0.02 at 734 nm. A mixture of 2 mL of the diluted radical solution and 20 μL of the extract was used to measure absorbance after 6 min with a spectrophotometer. The results were calculated based on a calibration curve built with Trolox standards and expressed as mg of Trolox equivalents (TE) per gram of DW. All assays were performed in triplicate.

### 2.7. HPLC-DAD Analysis of Phenolic Compounds

The phenolic compounds present in the extracts were separated and identified by using HPLC equipment (Hewlett-Packard 1100 series) furnished with a diode array detector programmed at different wavelengths for individual compounds and an Agilent 1100 series autosampler (20 μL samples were injected). The chromatographic column used was Kinetex EVO C18 100A of 5 μm particle size and dimensions of 250 × 4.6 mm of internal diameter from Phenomemenex^®^. The mobile phase consisted of HPLC Acetonitrile gradient grade 99.9% and milli-Q water with 0.01% in trifluoroacetic acid (TFA) (A). The flow rate was maintained at 1 mL/min and the chromatograms were recorded at wavelengths of 254, 280 and 340 nm. Linear gradient conditions for separation were as follows: 5% B (0–30 min); 25% B (30–45 min); 50% B (45–47 min); 100% B (47–50 min); 25% B (50–52 min); 5% B (52–55 min). The limit of detection (LOD) was 40 ng/mL and the limit of quantification (LOQ) was 50 ng/mL. All measurements were made in duplicate. The identification of the compounds was carried out by comparing their retention times and UV-visible spectrum at the wavelength characteristic of each compound and those of external standards. Figure S2 shows the chromatograms of the phenolic standards employed. Elenolic acid derivatives were quantified and expressed as oleuropeins.

### 2.8. Determination of Antimicrobial Activity

The antimicrobial activity of the extracts was investigated against the pathogens cited above through the minimum inhibitory concentration (MIC) and minimum bactericidal concentration (MBC) tests.

The MIC of the extracts was determined using a broth microdilution assay, following the standards for antimicrobial susceptibility testing provided by the Clinical and Laboratory Standards Institute from US (CLSL) [28,29]. The extract samples were prepared at concentrations ranging from 2.5 to 60 mg/mL in CAMHB (cation-adjusted MHB) and sterilized by filtration through a 0.22 µm filter (Filter-Lab, Barcelona, Spain). Microplate wells were filled with a volume of 200 µL containing approximately 5 × 10^5^ CFU/mL of test bacteria and variable concentrations of the extract prepared in CAMHB. Two sterility controls were prepared, one with the CAMHB medium and another with the extract. In addition, a negative control was prepared for inoculating the bacterial suspension in CAMHB medium. Microplates were introduced into a microplate absorbance reader (Bioscreen C Microbiology Reader, Oy Growth Curves Ab Ltd., Helsinki, Finland) and were incubated at 37 °C for 24 h, except for *Yersinia enterocolitica* wells, which were incubated for 48 h. Absorbance readings were set every hour at a wavelength of 600 nm. All assays were performed in triplicate. The MIC value corresponded to the lowest extract concentration at which no bacterial growth was visible. For this, cultures from each negative well (no turbidity) from the MIC assay were surface-plated on the appropriate medium, as explained in Section 2.2 [30].

### 2.9. Statistical Analysis

One-way analysis of variance (ANOVA) with Tukey’s post hoc test for pairwise multiple comparison was carried out, and Pearson’s correlation coefficient was calculated using the IBM^®^ SPSS^®^ Statistics Version 25 (IBM Corporation, New York, NY, USA). Significant differences were considered at a level of *p* < 0.05. All data were reported as mean ± standard deviation.

## 3. Results and Discussion

### 3.1. Influence of the Extraction Method on Total Phenol Content (TPC) and Antioxidant Activity (AA)

#### 3.1.1. Soxhlet Extraction Method

**Figure 1:**
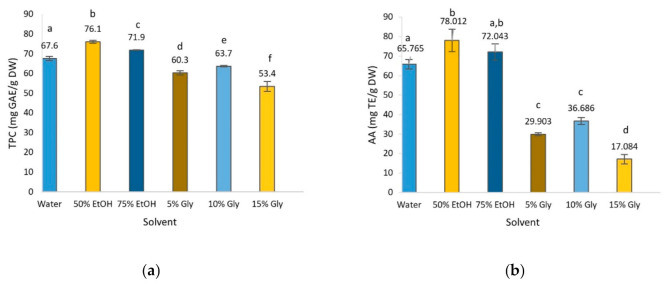
Total phenol content (TPC) (**a**) and antioxidant activity (AA) (**b**) of olive leaf extract obtained by Soxhlet extraction as a function of solvent. Different letters above the bars represent significant differences at *p* < 0.05.

The selection of an appropriate solvent is one of the most relevant issues in maximizing the recovery of plant phenols. In this work, the TPC and AA of olive leaf extract obtained using water, 50% EtOH, 75% EtOH, 5% glycerol, 10% glycerol and 15% glycerol, which are environmentally friendly, low-cost and non-toxic, were evaluated [12,13,31]. The TPC in olive leaf extracts presented in Figure 1a show that the highest concentration corresponded to 50% ethanol, followed by water and 75% ethanol; 5 and 10% glycerol showed a lower phenolic content with 15% glycerol exhibiting the lowest. The effect of the solvent was significant on the extraction of phenolic compounds, as can be observed in Figure 1a. In general terms, other studies reported values around 46% lower than those of the present work with the exception of the high content found by Da Rosa et al. [32] in 40% ethanolic extract [32,33,34]. Procedural factors such as the time of maceration with the solvent or other factors such as the degree of maturation of the leaves may account for these differences.

AA is the most studied bioactivity in plant extracts and has been attributed to the presence of certain bioactive compounds, mainly polyphenols. Significant differences were observed for the different solvents used (Figure 1b). AA values followed the same pattern as TPC values, where 50% EtOH was the most effective solvent, followed in decreasing order by 75% EtOH > water > 10% glycerol > 5% glycerol > 15% glycerol. These results are in agreement with those published by other authors, who reported that ethanol and water mixtures yielded extracts with higher AA than solely water or pure ethanol [7,31].

As mentioned above, AA is related to the presence of phenolic compounds, hence there should be a significant correlation between the concentration of polyphenols and antioxidant capacity, suggesting that these compounds contributed greatly to the antioxidant properties. In this study, Pearson’s correlation coefficient (r) was calculated to explain the relationship between TPC and AA values [35], finding a strong positive correlation between TPC and AA (r = 0.950). Moreover, it is widely accepted that other minor components such as volatile oils, carotenoids and vitamins probably also contribute to the AA of the extract [36]. These results are in concordance with several studies that reported a high correlation between polyphenolic compounds and AA [7,37,38].

#### 3.1.2. Microwave-Assisted Extraction (MAE) Method

MAE has been used as an alternative method for the recovery of plant extracts due to its reduced extraction time, higher extraction efficiency, less labor required and high extraction selectivity [8,9]. There are multiple parameters that affect the extraction efficiency of the MAE method such as solvent and composition, microwave temperature and extraction time [39]. Based on previous studies by other authors [40], who evaluated the effect of a wide range of temperatures and times on the recovery of phenolic compounds, the following values were selected on the basis of their cost-effective performance: temperature (40, 60 and 80 °C), time (3, 6.5 and 10 min) and solvent (the same as for Soxhlet extraction). TPC and AA of the extracts were measured under the different conditions (Tables S1 and S2).

**Figure 2:**
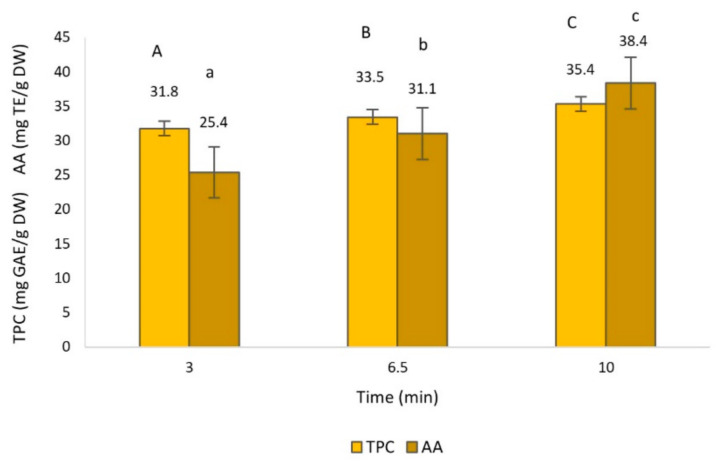
Total phenol content (TPC) and antioxidant activity (AA) of olive leaf extract obtained by microwave-assisted extraction (MAE) as a function of treatment time. Different capital and lowercase letters above the bars represent groups significantly different at *p* < 0.05 of TPC and AA, respectively.

**Figure 3:**
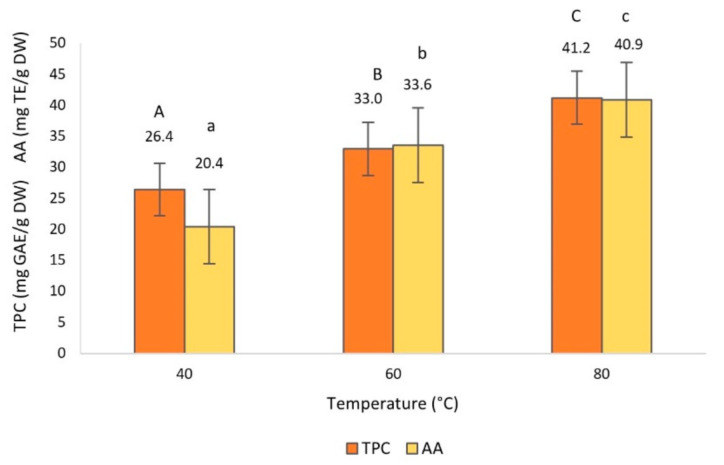
Total phenol content (TPC) and antioxidant activity (AA) of olive leaf extract obtained by microwave-assisted extraction (MAE), as a function of the extraction temperature. Different capital and lowercase letters above the bars represent groups significantly different at *p* < 0.05 of TPC and AA, respectively.

**Figure 4:**
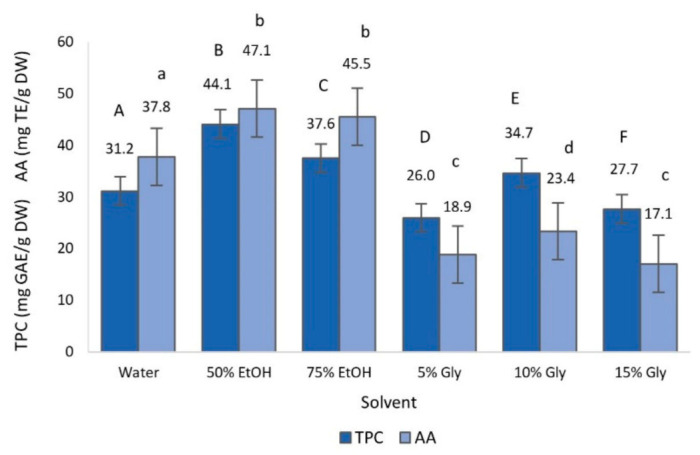
Total phenol content (TPC) and antioxidant activity (AA) of olive leaves extract obtained by microwave-assisted extraction (MAE), as a function of the type of solvent. Different capital and lowercase letters above the bars represent groups significantly different at *p* < 0.05 of TPC and AA, respectively.

The effect of the extraction time, temperature, solvent and their interactions on TPC and AA was statistically significant (*p* < 0.05), as can be appreciated in Figure 2, Figure 3 and Figure 4.

Regarding the extraction time, as expected, significantly greater TPC and AA at longer extraction times were observed (Figure 2). Similarly, in previous work with olive leaf extract, the same pattern was observed, in which the increase of extraction time, up to 10 min, had a positive influence on the recovery of TPC and AA [41,42]. In addition, it has been demonstrated by several authors that extraction times of longer than 10 min could bring no better results in TPC, and even decrease after 15 min [42], suggesting that long processing times may lead to the decomposition of phenolic compounds [32].

The effect of extraction temperature on TPC and AA is shown in Figure 3, where data reveal significant increments when temperature increases. Temperature is one of the main factors contributing to the efficiency of the MAE method [43]. Our results are entirely consistent with the findings of other authors who demonstrated that the TPC and AA values increased at high temperatures [11,32,40,41,43,44,45]. This positive correlation is explained by the fact that as the extraction temperature increases, the rate of diffusion and mass transfer of phenolic compounds into the solvent also does [11,32,41]. However, previous studies have shown that above 80 °C, the extraction efficiency declined due to thermal degradation of some phenolic compounds [40].

The use of different extraction solvents (Figure 4) resulted in significant differences in TPC, in which the 50% ethanolic extract presented the highest TPC value, followed by the 75% ethanolic extract. With regards to AA, no significant differences were observed between the 50 and 75% ethanolic extracts, presenting the highest AA values, and between 5 and 15% glycerol, with the lowest AA values. These results are in line with the study of Rafiee et al. [42], in which the 50% ethanolic extract showed the highest recovery of polyphenols in MAE of olive leaves. Likewise, Da Rosa et al. [32] reported that 40 and 70% ethanolic extracts showed greater AA values than water extract obtained by MAE from olive leaves.

The selection of a suitable solvent in MAE is one of the most relevant steps towards optimizing the recovery of phenols from plants and it is strongly affected by factors such as polarity, dielectric constant and viscosity of the solvent [42,46]. Although pure water is the most polar solvent, its high viscosity compared to the 50% ethanol solvent negatively affects the mass transfer and thus the extraction capacity. Therefore, the 50% ethanol solvent, having a lower viscosity, increases the swelling of the plant materials and the contact surface between the plant matrix and the solvent, enhancing the extraction yield [42,47]. However, the highest viscosity corresponds to glycerol solvents, which cause a slower external diffusion, thus reducing the extraction yield [11].

#### 3.1.3. Comparison of Soxhlet Method and MAE

Based on our results, for the sake of comparison of extraction methods, the most optimal combination of temperature and time of the MAE method was selected: 80 °C and 10 min (MAE-10-80) (see Figure 2 and Figure 3).

**Figure 5:**
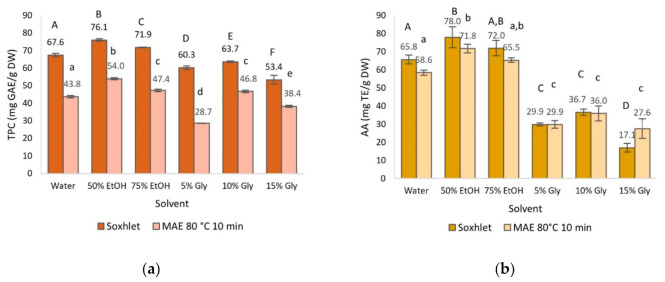
Comparison of (**a**) total phenol content (TPC) and (**b**) antioxidant activity (AA) of extracts obtained from Soxhlet and MAE at 80 °C for 10 min. Different capital and lowercase letters above the bars represent groups significantly different at *p* < 0.05 by Soxhlet and MAE-10-80 methods, respectively.

Regarding TPC, 50% ethanol was the most efficient solvent in both methods, whereas 15% glycerol in Soxhlet and 5% glycerol in MAE-10-80 resulted in the lowest TPC. As can be seen in Figure 5a, the type of solvent had a significant influence on TPC in the Soxhlet method while in MAE-10-80, the same applied with the exception of the 10% glycerol extract, whose TPC value was not statistically different from the 75% ethanolic extract. In general, it is observed that the TPC values obtained from the Soxhlet extracts were significantly higher than in those from MAE-10-80, although both methods followed the same trend. In fact, a positive linear correlation among the TPC in both methods was found (*r*^2^ = 0.747).

With reference to AA (Figure 5b), the highest and lowest values corresponded to the same extracts as for TPC, i.e., 50% ethanolic and 15% glycerol extracts, respectively. All in all, the same trend was observed as for the TPC value, although it should be mentioned that in the case of MAE, the AA values from the three glycerol solvents did not present significant differences. A strong correlation was found (*r*^2^ = 0.970) between AA values of extracts from Soxhlet and MAE-10-80 methods. In addition, ANOVA results indicated that no significant differences were observed between both extraction methods in every extract tested.

The correlation value obtained for TPC, weaker than the AA correlation, suggests that the TPC values determined by Soxhlet may have been overestimated. This could be because the high presence of impurities in Soxhlet extracts, such as organic acids, sugars and proteins, could interfere with the quantification of phenolic compounds by reacting with the Folin–Ciocalteu reagent, thus causing an overestimation of the measurement [42,45,48].

Our results and the characteristics of the extraction methods drive us to consider MAE as a suitable alternative to Soxhlet because of its efficiency in the recovery of phenolic compounds even when applied for a short time [32,42]. Indeed, in MAE, the interaction between microwaves and the solvent molecules causes the temperature and internal pressure of the plant product to increase rapidly, resulting in an intense rupture of the plant cell wall, which leads to a faster release of the cell compounds into the solvent [32] and, therefore, to a higher extraction yield [43]. In relation to the solvent employed, our study shows that 50% ethanol would be the solvent with the best performance due to its higher efficacy, reduced cost, and toxicity [42]. However, it should be highlighted that water deserves special attention because minor differences with ethanolic extracts were encountered in the present study, where TPC and AA of the 50% ethanolic extracts were only 1.12–1.23 times higher than those of water extracts (Figure 5). Consistent with these findings, other authors have reported comparable TPC values in both water and ethanolic extracts [7,31,32,43,44]. In relation to glycerol, despite having potential to be used as a green solvent in MAE [43], irradiation time should be increased to obtain major recovery of polyphenols [34].

### 3.2. Identification and Quantification of Phenolic Compounds by HPLC

HPLC analysis was carried out on the extracts obtained by Soxhlet extraction and MAE-10-80. Identification and quantification of seven phenolic compounds by HPLC is shown in Table 1: one simple phenol (hydroxytyrosol), four flavonoids (luteolin, luteolin-7-O-glucoside, apigenin and apigenin-7-O-glucoside), one secoiridoid (oleuropein) and one cinnamic acid derivative (verbascoside) [39]. The mentioned polyphenols have been previously reported by several authors in different olive leaf extracts [16,44,49,50,51,52].

The one-way ANOVA test revealed that the type of method, solvent and the interaction of both factors had a significant effect on the total phenols (quantified as sum of individuals) identified by HPLC. Depending on the solvent used, the range of total phenols in the Soxhlet extraction varied between 4.82 and 37.22 mg/g DW, and in MAE-10-80 from 15.44 to 48.52 mg/g DW. Previously, other authors had described variable concentrations of total phenols in olive leaf extracts of the Hojiblanca variety, up to three times lower than ours [53,54], contrary to those published by Martín-García et al. [16] of up to three times higher. It is worth noting that these large variations in the composition of olive leaf extracts are likely due to issues such as the cultivar, solvent, extraction methodology, analytical method, as well as diverse abiotic factors (geographical origin, harvest time and light exposition) and biotic factors (genotypes and leaves age), among others [39,53,55].

In Soxhlet extraction, the highest amount of total phenols corresponded to the 75% ethanolic extract, while in MAE-10-80, it was the 50% ethanolic extract. It should be noted that the extraction method, due to the different extraction conditions, has a significant influence on the recovery of total polyphenols. In MAE, with a short extraction time (10 min) and a fixed temperature (80 °C), the 50% ethanol solvent achieved a slightly higher concentration of polyphenols than the 75% ethanol solvent. However, in Soxhlet, long extraction times were employed, which in combination with the higher temperature reached in the 50% ethanol extraction, could have led to a higher degradation of phenolic compounds than in the 75% ethanol extraction [56], with a lower temperature reached, and thus, a slightly higher recovery of polyphenols. These results are in agreement with other literature data that demonstrated that mixtures of ethanol solvents can lead to higher total phenols content compared to water [57]. In contrast, the work reported by Apostolakis et al. [34] revealed that water–glycerol mixtures extracted more polar compounds than those found in water–ethanol mixtures, an event not observed in our study. With reference to the different ethanol mixtures, diverse results have been found in the literature [58], not concluding with a definitive universal optimum ethanol mixture.

Likewise, MAE showed a higher value of total phenols than the Soxhlet method (*p* < 0.05), and this finding was also documented by other authors, who have reported that the extraction method influences the quantity of total phenols, demonstrating that MAE extracts achieved a better recovery of total phenols and oleuropein than the other extraction methods [40,55,59]. Despite these facts, the Pearson’s correlation coefficient indicated a significant positive correlation between both methods (*r*^2^ = 0.894), suggesting that the ‘response’ of phenolic compounds to both extraction methods followed a similar trend for the different solvents tested.

On the other hand, comparing the total phenols by HPLC with the TPC determined by the Folin–Ciocalteu method (Table 1 and Figure 5a, respectively), a slight difference was observed, the TPC value being higher than the sum of phenolic compounds quantified by HPLC. This difference could be explained, as commented previously, by the fact that the Folin–Ciocalteu reagent can react with other non-phenolic substances [48] and by the presence of non-identified/quantified compounds by HPLC analysis. Despite these differences, a high correlation was obtained between TPC by Folin–Ciocalteu and total phenols by HPLC in both extraction methods, MAE-10-80 (*r*^2^ = 0.847) and Soxhlet (*r*^2^ = 0.812).

In accordance with other authors in previous research [33,39,60], oleuropein and hydroxytyrosol were the most abundant compounds in olive leaf extracts (see Table 1). In contrast, the minor compound was apigenin-7-O-glucoside. Significant differences in the content of oleuropein, hydroxytyrosol, verbascoside, luteolin, luteolin-7-O-glucoside and apigenin were also noted depending on the method and solvent used in the extraction.

As widely demonstrated, the main family of compounds present in olive leaves are the secoiridoids, oleuropein, constituted by hydroxytyrosol and elenolic acid, being the major phenolic compound [17,52,60]. The range of oleuropein content (Table 1) oscillated in a wide range, between 1.05 and 40.49 mg/g DW. MAE-10-80 showed the highest oleuropein values compared to Soxhlet (*p* < 0.05), with results in accordance with those of Taamalli et al. [40], who reported a significantly higher recovery of the main secoiridoids in MAE olive leaf extracts. In general, it can be seen that the trend of oleuropein recovery as a function of the solvent used followed the same pattern as TPC recovery, the 50 and 75% ethanolic extracts being the ones that yielded the highest oleuropein value [57,61,62]. 

Hydroxytyrosol, the main degradation product of oleuropein, is the second major component of the olive leaf extracts studied, behind oleuropein [39]. The highest amount of hydroxytyrosol determined in this work was within the range of other studies (0.3–11.4 mg/g DW) [60]. Unlike oleuropein, the Soxhlet method was more efficient in hydroxytyrosol extraction, except for 15% glycerol. This may be explained by the fact that the Soxhlet method, which involves higher temperature and longer extraction time, leads to a greater degradation of oleuropein in the course of the treatment, resulting in a higher concentration of hydroxytyrosol [17]. The hydroxytyrosol contents obtained by both methods were similar to those reported by Herrero et al. [54] for the same variety (Hojiblanca), in spite of using different methods and solvents.

Verbascoside has been reported in literature at concentrations of up to 29 mg/g DW [60]. In the extracts analyzed in this work, our maximum value (1.48 mg/g DW) was approximately fifteen times lower than those reported by Ahmad-Qasem et al. [45], but in contrast, the minimum (0.12 mg/g DW) was similar to data cited by Japón-Luján et al. [63]. With regards to luteolin-7-glucoside, its concentration was lower than its aglycon form (luteolin), contrary to the common findings in olive leaves [50,60]. The concentration of both forms of luteolin followed the same trend, with the highest concentrations in the 50 and 75% ethanolic extracts [54,57]. Similar to luteolin and luteolin-7-glucoside, several studies have reported higher values of apigenin-7-glucoside than apigenin, in contrast with our results, where apigenin-7-glucoside was found at trace levels [54,60]. An issue already mentioned in this work, that should be emphasized, is the fact that multiple variables may affect the polyphenol profile of olive leaf extracts, justifying the great quantitative and qualitative differences reported in literature.

All in all, our work shows that the extracts analysed have a valuable content of polyphenols, the two extraction methods studied being adequate and efficient. Specifically, MAE-10-80 provided a higher yield of oleuropein probably due to its low degradation through short times and low temperatures of extraction. In general, the extracts presented high values of oleuropein and hydroxytyrosol, especially the ethanolic extracts, both compounds being widely used in the food, nutraceutical, cosmetics and pharmaceutical industries [53]. Although there are multiple factors that may affect the composition of the extracts [45], in light of our results with respect to total phenols and AA, and taking into account the features of the methods, MAE was selected for subsequence antimicrobial assays [55,59].

### 3.3. Antibacterial Properties

#### 3.3.1. Antimicrobial Activity of Olive Leaf Extracts and Solvent Efficacy

It is well-known that the antimicrobial properties of plant extracts have been attributed to phenolic compounds [14,16,17,18]. To evaluate this antimicrobial activity, three extracts obtained by MAE were selected, corresponding to different solvents and based on the major AA achieved and minimum solvent concentration. The antimicrobial tests performed, in terms of MIC and MBC, demonstrated a similar degree of inhibition for the three solvents used, i.e., water (MAE-W), 50% ethanol (MAE-Et50) and 5% glycerol (MAE-Gly5), as can be observed in Table 2, where MIC and MBC concentrations were, in general terms, at the same level for all microorganisms assayed, i.e., 20–60 mg/mL, with the exception of MAE-W in *S. aureus* and *Y. enterocolitica*, with MIC-MBC values of 2.5–5 mg/mL and 5–10 mg/mL, respectively. However, looking at the MIC and MBC figures, slight differences in solvent efficacy may shape a trend, that is, in decreasing order of antimicrobial activity, MAE-W > MAE-Et50 > MAE-Gly5, except for *E. coli* for which MAE-Et50 was slightly more successful than MAE-W.

With regards to the microorganisms tested, a sensitivity rank can also be withdrawn from Table 2, i.e., *S*. *aureus* > *Y. enterocolitica* > *L. monocytogenes* > *E. coli* > *S*. *Typhimurium*. In fact, the inhibition curves, showing the kinetic behavior of microorganisms in the presence of the extracts tested (MAE-W, MAE-Et50 and MAE-Gly5) at the MIC and MBC concentrations and in the absence of extracts, corroborate the MIC and MBC established (Figure S1).

Different studies have addressed the antimicrobial activity of olive leaf extracts against specific microorganisms. Gullón et al. [7] found very similar values to ours in 50% ethanolic extracts of olive leaves and olive pruning. For example, for *E. coli* and *S. enterica* subsp. enterica, very similar MIC and MBC values were reported (30–45 mg/mL); in the case of *S. aureus*, the extract showed a MIC and MBC between 20–30 mg/mL, close to the range of our results for MAE-Et50 and MAE-Gly5, while for *L. innocua,* slightly lower values were reported (20–25 mg/mL). Furthermore, in accordance with our study, Liu et al. [64] demonstrated that at 62.5 mg/mL, ethanolic extracts of olive leaves (80% ethanol; solid–liquid extraction) almost completely inhibited the growth of *L. monocytogenes*, *E. coli* O157:H7, and *Salmonella enteritidis*. One year later, the same research group reported a MIC value of 62.5 mg/mL for *L. monocytogenes*, and greater than 62.5 mg/mL (non-determined) for *E. coli* O157:H7 and *S*. Enteritidis in a commercial olive leaf extract [65]. In addition, Gökmen et al. [66] reported similar MIC values against *S. aureus*, *E. coli*, *L. monocytogenes* and *S*. Typhimurium, between 32 and 64 mg/mL. Similarly, Techathuvanan et al. [67] reported a range of MIC values from 2 to 2.5 mg/mL against *S. aureus* in a commercial olive leaf extract comparable to our MIC value in MAE-W extract, while in the work published by Şahin et al. [68], *S. aureus* showed higher sensitivity when tested in an aqueous extract obtained by MAE (MIC = 1.25 mg/mL).

However, other studies have reported variable MIC and/or MBC values, which deviated from our results. In the case of *E. coli*, lower values of MIC and MBC (of 1.25 and 2.5 mg/mL, respectively) were achieved in a 70% ethanolic extract by solid–liquid extraction [69], while Pereira et al. [70], using water as solvent, found that 1.81 mg/mL of their extract inhibited 25% of microbial growth. Masoko and Makgapeetja [71], despite finding the same MIC value (2.5 mg/mL) of water extract (solid–liquid extraction) for *S. aureus* as the MIC of the present study, in the case of ethanolic extracts, the opposite to our results with MAE-Et50, found a very low MIC value (0.26 mg/mL). Regarding *Salmonella* spp., Hemeg et al. [69] tested the antimicrobial activity of olive leaf 70% ethanolic extract by solid–liquid extraction, finding MIC and MBC values of 2.5 and 5 mg/mL, respectively. Techathuvanan et al. [67] found that a commercial olive extract had antimicrobial activity against *L. monocytogenes*, with a MIC value ranging from 2.2 to 2.6 mg/mL, similar to that observed by Testa et al. [72]. With respect to *Y. enterocolitica*, although yersiniosis was the fourth most reported zoonosis in humans in the EU in 2019 [73], only a few studies have evaluated the antimicrobial activity of olive leaf extracts against this pathogen. Medina-Martinez et al. [74] observed in a commercial hydroxytyrosol extract MIC values higher than 1 mg/mL (non-determined) for *Y. enterocolitica*.

As it has been observed, scientific evidence shows a considerably wide range of MIC and MBC values of olive leaf extracts against bacterial foodborne pathogens. It may be explained by variations in the strains sensitivity, by the method followed for antimicrobial assays, by the phenolic composition of the extracts, as well as other issues such as the extraction procedure, type of solvent and tree variety [7,75]. Furthermore, it is noted that there is not a definite trend in the sensitivity of microorganisms to the extracts as a function of their wall characteristics; indeed, the most sensitive microorganisms tested in our study, i.e., *S. aureus* and *Y. enterocolitica*, are Gram-positive and Gram-negative, respectively. Some authors argue that Gram-negative bacteria are deemed as more resistant due to the absence of a lipopolysaccharide layer in their wall, present in Gram-positive bacteria, which makes Gram-negative bacteria more impermeable to antimicrobial compounds [7,64,76]. However, other works have reported different results [77,78], it being impossible to delineate a characterization of Gram-negative and Gram-positive bacteria with respect to their sensitivity to these extracts [79]. All in all, it can be concluded that olive leaf extracts induce a strong antimicrobial action against some of the most common agents implicated in bacterial foodborne diseases, as demonstrated in this study [73].

Our results show a very promising application of water as a universal molecule to obtain olive leaf extracts with an enhanced antibacterial activity (MAE-W) in comparison with the extracts obtained with solvent mixtures such as water–ethanol 50:50% (MAE-Et50) or water–glycerol 95:5% (MAE-Gly5). Other authors, however, have reported lower antimicrobial activity of aqueous olive leaf extract (solid–liquid extraction) against *S. aureus* and *E. coli* than ethanolic extracts [71]. In our study, except for *E. coli*, with a slightly enhanced inhibition by MAE-Et50, for the rest of the pathogens tested, water was the solvent of choice, in the light of the superior antibacterial efficacy of MAE-W (see Table 2) and bearing in mind important economic, environmental and safety issues.

#### 3.3.2. Influence of Phenolic Composition of MAE Extracts Obtained with Water, 50% Ethanol and 5% Glycerol Solvents on Antimicrobial Activity

**Figure 6:**
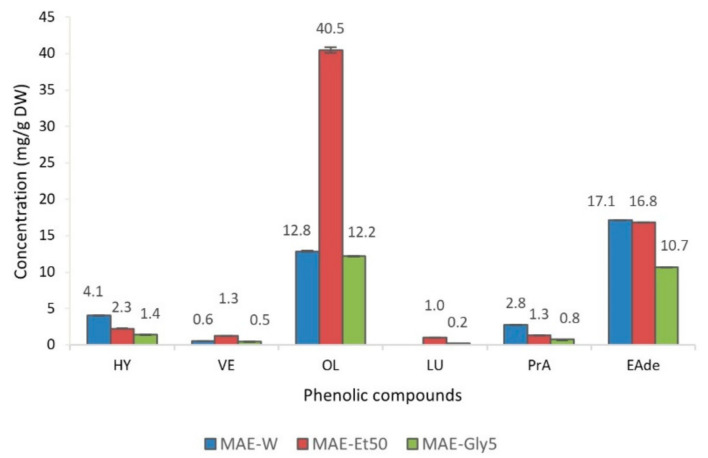
Concentration (mg/g dry weight) of phenolic compounds in microwave-assisted extraction (MAE) extracts, using water (MAE-W), 50% ethanol (MAE-Et50) and 5% glycerol (MAE-Gly5) as solvents. Abbreviations: HY, hydroxytyrosol; VE, verbascoside; OL, oleuropein; LU, luteolin; PrA, protocatechuic acid; EAde, elenolic acid derivatives (sum of 3 derivatives compounds).

Figure 6 shows the concentration of different phenolic compounds of MAE-W, MAE-Et50 and MAE-Gly5 extracts. Additional phenolic compounds, i.e., elenolic acid derivatives or protocatechuic acid, were quantified based on their sound antimicrobial activity (Figures S2 and S3) [79]. The high concentration of oleuropein found in MAE-Et50 stands out, with around one third of it in the case of the other two extracts; on the contrary, MAE-W contains a higher concentration of other relevant phenolic compounds such as hydroxytyrosol, elenolic acid derivatives or protocatechuic acid.

Many authors have attributed to oleuropein the antimicrobial activity of olive leaf extracts, mainly based on the fact that, traditionally, oleuropein has been the major compound encountered, and thus, the one which has received more attention over the last decades [49,70,80,81,82]. However, as can been observed in Table 2, this hypothesis cannot be corroborated by our study, as MAE-Et50 was not the extract showing the best antimicrobial performance, but water. Thielmann et al. [79], supported by previous research studies [83,84], stated that elenolic acid derivatives, although scarcely investigated, are the compounds presenting the strongest antimicrobial activity. These conclusions support the results obtained in this study, as the extract with the highest content of elenolic acid derivatives and other minor substances (e.g., hydroxytyrosol or protocatechuic acid) was MAE-W, the most successful antibacterial extract in this study. Hydroxytyrosol, part of the oleuropein molecule together with elenolic acid, possesses a demonstrated stronger inhibitory capacity than oleuropein [75]; already in 1999, Bisignano et al. [85] determined a MIC for hydroxytyrosol around 32 times and 8 times less than the MIC established for oleuropein against *Salmonella typhi* ATCC 6539 and *S. aureus* ATCC 25923, respectively.

Nevertheless, in the case of complex mixtures of bioactive compounds, it is very difficult to assign the antimicrobial activity to specific components [7]. In this sense, it is highly relevant to consider that the antimicrobial activity of extracts is not only due to their chemical composition and the mechanism of action of their bioactive constituents, but also the interaction (synergism, antagonism, chemical reactions) between them, and between these and other substances such as culture medium nutrients [69,86]. The interaction between phenolic compounds, and specifically the synergistic phenomenon, has been observed by several authors, leading to stronger antibacterial activities [70,87]. More research is needed to elucidate these facts and their associated mechanisms, which may help to clarify the contradictory results of antimicrobial effects on Gram-positive and -negative bacteria. Some mechanisms attributed to polyphenolic compounds have been proposed, including protein denaturation, inhibition of enzymatic reactions necessary for bacterial growth and increase of cell membrane permeability; these are able to interfere with the structural and functional properties of bacterial membranes by interacting with cell membrane lipids, causing the leakage of cytoplasmic contents [7,85,88,89].

Despite the excellent performance of the extracts obtained, especially MAE-W, against the foodborne pathogens considered, some issues should be borne in mind for practical applications. Medina et al. [90] reported that phenolic compounds exhibit antimicrobial activity against beneficial bacteria for health, such as *Lactobacillus acidophilus* and *Bifidobacterium bifidum.* However, opposite to this idea, several studies have stated that the prebiotic activity of polyphenols enhances the growth of beneficial bacteria (Bifidobacterium and Lactobacillus, among others), while acting as antimicrobials against pathogenic bacteria (*E. coli*, *Clostridium perfringens* and *Helicobacter pylori*) by reducing their nutrient availability [59,91,92].

## 4. Conclusions

Olive industry by-products, such as olive leaves, constitute a natural resource of valuable compounds that could enter the production and economy cycle, thus taking care of sustainability, environmental, market and socioeconomic issues, in pursuit of the so-called bioeconomy. Olive leaf waste has a huge added-value potential, mainly attributed to its content in phenolic compounds, with demonstrated antioxidant and antimicrobial activity. Two extraction methods, Soxhlet and MAE, were assayed to evaluate their performance on phenol extraction, and subsequently, their bioactivity. Although Soxhlet achieved the best extraction of phenolic compounds (TPC) in comparison with MAE, this cannot be extrapolated to the antioxidant activity (AA), with comparable results in both methods. For this reason, MAE is proposed as an optimum alternative to the detriment of the conventional Soxhlet method, also entailing additional benefits such as low energy cost, short process time (10 min versus 5 h in Soxhlet) and low degradation of bioactive compounds. Regarding the type of solvent employed, the results show that the 50% ethanolic solution was the solvent with the best extraction performance; and indeed, the TPC and AA in 50% ethanolic extracts were found to be slightly higher (with a factor of 1.12–1.23) than in water extracts. However, despite this observation, the pathogens tested in this study showed higher sensitivity to water extract than to the others. With regards to glycerol solvent, notwithstanding its potential for phenol extractions reported by some authors, a long irradiation time in MAE is needed to obtain competitive results in comparison with ethanolic and water solvents.

Olive leaf MAE-W extract is rich in elenolic acid derivatives and other phenolic compounds with a strong antimicrobial activity such as hydroxytyrosol, conferring a great potential to this extract for its application in the food, cosmetic and pharmaceutical industries. Although oleuropein is a well-known and characterized molecule in olive leaf extracts, being present at high levels especially in ethanolic ones, it has been demonstrated that it is not the main actor in the bioactivity, namely, antioxidant and antimicrobial activity, of the MAE extracts. This study demonstrates that water, as a universal, safe and cheap solvent, applied to obtain MAE extracts from olive leaves with antioxidant and antibacterial activity, could become the ultimate link to close the bioeconomy circle.

## Figures and Tables

**Table 1:** Concentration (mg/g DW) of the main phenolic compounds identified in olive leaf extracts obtained by Soxhlet and MAE 10-80 (microwave-assisted extraction at 80 °C for 10 min) with different solvents analyzed by HPLC. Chromatograms are available in Figure S3.

Extraction Method	Solvent	Phenolic Compounds							
		HY	VE	LU-7	OL	LU	AP	AP-7	Total
**Soxhlet**	Water	4.68 ± 0.14 ^a^	1.48 ± 0.18 ^a^	Traces	6.95 ± 0.07 ^a^	0.02 ± 0.00 ^a^	0.50 ± 0.06 ^a^	Traces	13.99 ± 0.50 ^a^
	50% EtOH	8.04 ± 0.32 ^b^	0.71 ± 0.08 ^b^	1.55 ± 0.16 ^a^	18.44 ± 0.92 ^b^	1.55 ± 0.15 ^b^	0.44 ± 0.03 ^a,b^	Traces	30.74 ± 1.66 ^b^
	75% EtOH	5.13 ±0.11 ^a^	0.54 ± 0.02 ^b^	1.83 ± 0.13 ^b^	27.13 ± 2.22 ^c^	1.79 ± 0.08 ^c^	0.45 ± 0.14 ^a,b^	Traces	37.22 ± 2.83 ^c^
	5% Gly	1.67 ± 0.13 ^c^	0.65 ± 0.08 ^b^	0.53 ± 0.11 ^c^	1.05 ± 0.27 ^d^	0.50 ± 0.02 ^d^	0.27 ± 0.05 ^b^	Traces	4.82 ± 0.71 ^d^
	10% Gly	5.97 ± 0.32^d^	0.16 ± 0.06 ^c^	Traces	9.74 ± 0.68 ^a^	0.08 ± 0.00 ^a^	0.04 ± 0.00 ^c^	Traces	15.99 ± 1.06 ^a^
	15% Gly	0.50 ± 0.06 ^e^	0.12 ± 0.00 ^c^	Traces	8.67 ± 0.86 ^a^	0.02 ± 0.00 ^a^	0.003 ± 0.00 ^c^	Traces	9.35 ± 0.92 ^e^
**MAE 10-80**	Water	4.06 ± 0.02 ^a^	0.56 ± 0.01 ^a^	Traces	12.84 ± 0.07 ^a^	Traces	Traces	Traces	18.60 ± 0.10 ^a^
	50% EtOH	2.28 ± 0.01 ^b^	1.27 ± 0.01 ^b^	0.51 ± 0.00 ^a^	40.49 ± 0.43 ^b^	1.02 ± 0.01 ^a^	0.38 ± 0.01 ^a^	Traces	48.52 ± 0.50 ^b^
	75% EtOH	1.91 ± 0.01 ^c^	1.06 ± 0.01 ^c^	0.52 ± 0.00 ^b^	38.92 ± 0.71 ^b^	0.67 ± 0.01 ^b^	0.25 ± 0.01 ^b^	Traces	44.05 ± 0.75 ^c^
	5% Gly	1.40 ± 0.01 ^d^	0.48 ± 0.00 ^d^	Traces	12.19 ± 0.01 ^a^	0.21 ± 0.00 ^c^	Traces	Traces	15.44 ± 0.03 ^d^
	10% Gly	2.87 ± 0.02 ^e^	0.97 ± 0.01 ^e^	Traces	27.80 ± 1.69 ^c^	0.58 ± 0.00 ^d^	0.29 ± 0.01 ^c^	Traces	34.97 ± 1.74 ^e^
	15% Gly	1.51 ± 0.01 ^f^	0.69 ± 0.00 ^f^	0.16 ± 0.00 ^c^	21,76 ± 1.65 ^d^	0.32 ± 0.00 ^e^	0.16 ± 0.01 ^d^	Traces	25.63 ± 1.68 ^f^

Values are expressed as mean ± standard deviation. A standard deviation value of ‘0.00’ indicates values between 0.0001 and 0.0039. Within each extraction method, different superscript letters in the same column indicate values significantly different (*p* < 0.05) according to Tukey’s Multiple Range Test. Abbreviations: HY, hydroxytyrosol; VE, verbascoside; LU-7, luteolin-7-O-glucoside; OL, oleuropein; LU, luteolin; AP, apigenin; AP-7, apigenin-7-O-glucoside; Total, sum of individuals. Traces: under LOD (limit of detection).

**Table 2:** Minimum inhibitory concentration (MIC) and minimum bactericidal concentration (MBC) of olive leaf extracts against five food pathogens strains.

Bacterial Strains	Solvent	MIC (mg/mL)	MBC (mg/mL)
*Staphylococcus aureus* (CECT 5193)	MAE-W	2.5	5
	MAE-Et50	20	30
	MAE-Gly5	20	30
*Salmonella* Typhimurium (CECT 704)	MAE-W	40	60
	MAE-Et50	40	50
	MAE-Gly5	60	>60
*Escherichia coli* (CECT 8295)	MAE-W	40	50
	MAE-Et50	30	40
	MAE-Gly5	60	>60
*Listeria monocytogenes* (CECT 4032)	MAE-W	30	40
	MAE-Et50	40	50
	MAE-Gly5	>60	>60
*Yersinia enterocolitica* (CECT 754)	MAE-W	5	10
	MAE-Et50	20	30
	MAE-Gly5	20	30

## Data Availability

The data presented in this study are available on request from the corresponding author.

## Supplementary Materials

The following are available online at https://www.mdpi.com/article/10.3390/foods10050966/s1, Table S1: Total phenol content (TPC) (mg GAE/g DW) of olive leaf extracts by microwave-assisted extraction (MAE). Table S2: Antioxidant activity (AA) (mg TE/g DW) of olive leaf extracts by microwave-assisted extraction (MAE). Figure S1: Growth of (a) *S. aureus*, (b) *S.* Typhimurium, (c) *E. coli*, (d) *L. monocytogenes* and (e) *Y. enterocolitica* in broth with olive leaves extracts added. Figure S2: HPLC-UV chromatograms at 280 nm, the UV spectra and the retention time (Rt) of the phenolic standards employed to investigate and quantify these compounds in the phenolic extracts. Figure S3: HPLC-UV chromatograms at 280 nm of the phenolic extracts obtained by two methods and two solvents: (a) Soxhletwater; (b) Soxhlet50% EtOH; (c) microwave-assisted extraction at 80 °C during 10 min (MAE 10-80)water; (d) MAE 10-80-50% EtOH. Quantified compounds were: (1) hydroxytyrosol, (2) verbascoside, (3) luteolin-7-O-glucoside, (4) apigenin-7-O-glucoside, (5) luteolin, (6) oleuropein, and (7) apigenin.
