# Supplementary material for: Valorisation of Olea europaea L. Olive Leaves through the Evaluation of Their Extracts: Antioxidant and Antimicrobial Activity
Source: Foods. 2021 Apr 28;10(5):966. doi: 10.3390/foods10050966 (PMC8145053; doi:10.3390/foods10050966)
Supplement: Supplementary file 1 [file foods-10-00966-s002.zip › foods-1160599-supplementary.pdf]

Table S1. Total phenol content (TPC) (mg GAE/g DW) of olive leaf extracts by Microwave-Assisted Extraction (MAE).

**Time (min)**	**Temperature (°C)**	**Solvent**					
		Water	50% Ethanol	75% Ethanol	5% Glycerol	10% Glycerol	15% Glycerol
**3**	40	23.05 ± $0.08^{u,v}$	36.25 ± $0.26^{i,j}$	30.56 ± $0.17^{n,o}$	24.16 ± $0.47^{t,u}$	17.96 ± $0.43^{x,y}$	17.30 ± 0.07^{y}
	60	27.01 ± $0.83^{q,r}$	37.78 ± $0.38^{,h,i}$	34.17 ± $0.61^{k,l,m}$	25.87 ± $0.18^{r,s}$	34.49 ± $0.45^{k,l}$	31.77 ± $0.24^{m,n}$
	80	41.68 ± 0.36^{g}	45.60 ± $0.66^{c,d}$	41.31 ± 0.14^{g}	26.77 ± $0.82^{q,r}$	38.50 ± 0.54^{h}	35.05 ± 0.30^{h}
**6.5**	40	23.12 ± $0.42^{u,v}$	35.12 ± $0.31^{j,k,l}$	28.90 ± 0.42^{p}	18.85 ± $0.10^{w,x,y}$	28.10 ± $0.06^{p,q}$	25.41 ± $0.32^{r,s,t}$
	60	31.37 ± $0.08^{m,n}$	45.51 ± $0.29^{c,d}$	33.85 ± $0.33^{l,m}$	26.56 ± $0.58^{q,r}$	30.75 ± 0.81^{n}	24.66 ± $0.32^{s,t,u}$
	80	41.93 ± 0.93^{g}	53.19 ± 0.56^{a}	44.07 ± $0.30^{d,e}$	34.30 ± $0.85^{k,l,m}$	44.19 ± $0.57^{d,e}$	32.75 ± 1.14^{m}
**10**	40	19.82 ± 0.30^{w}	36.28 ± $0.12^{i,j}$	35.48 ± $0.26^{j,k}$	22.39 ± 0.47^{v}	34.21 ± $0.23^{k,l,m}$	18.94 ± $0.08^{w,x}$
	60	29.01 ± $0.07^{o,p}$	53.31 ± 0.44^{a}	42.23 ± $0.34^{f,g}$	26.19 ± $0.20^{r,s}$	37.13 ± $0.40^{h,i}$	22.17 ± 0.31^{v}
	80	43.80 ± $0.74^{e,f}$	54.05 ± 0.57^{a}	47.39 ± 0.74^{b}	28.72 ± 0.14^{p}	46.79 ± $0.64^{b,c}$	38.37 ± 0.61^{h}

Values are expressed as mean ± standard deviation. Different superscript letters indicate values significantly different ($p < 0.05$) according to Tukey´s Multiple Range Test.

Table S2. Antioxidant activity (AA) (mg TE/g DW) of olive leaf extracts by Microwave-Assisted Extraction (MAE).

**Time (min)**	**Temperature (°C)**	**Solvent**					
		Water	50% Ethanol	75% Ethanol	5% Glycerol	10% Glycerol	15% Glycerol
**3**	40	$13.21 \pm 1.97^{w,x,y,z}$	$13.78 \pm 0.98^{v,w,xyz}$	$24.28 \pm 1.89^{o,p,q,r,s,t}$	$12.02 \pm 0.64^{x,y,z}$	$13.26 \pm 1.17^{w,x,y,z}$	9.72 ± 0.92^{z}
	60	$30.28 \pm 1.72^{l,m,n,o}$	$33.33 \pm 1.54^{k,l,m}$	$40.10 \pm 1.35^{i,j,k}$	$16.18 \pm 1.70^{t,u,v,w,x,y,z}$	$19.88 \pm 1.84^{q,r,s,t,u,v,w,x}$	$15.37 \pm 1.45^{u,v,w,x,y,z}$
	80	$50.59 \pm 1.99^{d,e,f,g,h}$	$51.41 \pm 1.22^{d,e,f,g}$	$54.07 \pm 1.08^{c,d,e,f}$	$18.05 \pm 1.32^{s,t,u,v,w,x,y}$	$23.12 \pm 1.88^{o,p,q,r,s,t,u}$	$19.07 \pm 1.71^{r,s,t,u,vwxy}$
**6.5**	40	$17.28 \pm 1.23^{s,t,u,vwxyz}$	$42.94 \pm 1.44^{h,I,j}$	$28.66 \pm 0.97^{l,m,n,o,p}$	$12.32 \pm 1.62^{x,y,z}$	$14.53 \pm 1.47^{v,w,x,y,z}$	$11.00 \pm 1.37^{y,z}$
	60	$47.37 \pm 2.51^{f,g,h,i}$	$50.46 \pm 0.44^{d,e,f,g,h}$	$50.99 \pm 0.73^{d,e,f,g,h}$	$17.90 \pm 1.88^{s,t,u,v,w,x,y}$	$20.62 \pm 1.62^{p,q,r,s,t,u,v,w}$	$14.36 \pm 1.96^{v,w,x,y,z}$
	80	$52.64 \pm 1.71^{d,e,f,g}$	$58.27 \pm 0.86^{b,c,d}$	$57.04 \pm 0.15^{c,d}$	$19.93 \pm 1.41^{q,r,s,t,u,v,w,x}$	$27.04 \pm 1.88^{m,n,o,p,q,r}$	$15.64 \pm 1.84^{u,v,w,x,y,z}$
**10**	40	$21.66 \pm 1.78^{p,q,r,s,t,u,v}$	$45.59 \pm 0.78^{g,h,i}$	$27.95 \pm 1.96^{l,m,n,o,p,q}$	$19.05 \pm 1.58^{r,s,t,u,v,w,x,y}$	$23.17 \pm 2.11^{o,p,q,r,s,t,u}$	$16.84 \pm 2.02^{s,t,u,vwxyz}$
	60	$48.22\pm 2.26^{e,f,g,h,i}$	$56.18 \pm 2.20^{c,d,e}$	$61.40 \pm 1.68^{b,c}$	$24.94 \pm 1.91^{n,o,p,q,r,s}$	$32.69 \pm 2.46^{k,l,m,n}$	$23.92 \pm 2.61^{o,p,q,r,s,t}$
	80	$58.56 \pm 1.43^{b,c,d}$	71.81 ± 2.45^{a}	$65.45 \pm 1.24^{a,b}$	$29.86 \pm 2.13^{l,m,n,o}$	$36.03 \pm 3.18^{j,k,l}$	$27.59 \pm 2.33^{m,n,o,p,q}$

Values are expressed as mean ± standard deviation. Different superscript letters indicate values significantly different ($p < 0.05$) according to Tukey´s Multiple Range Test.

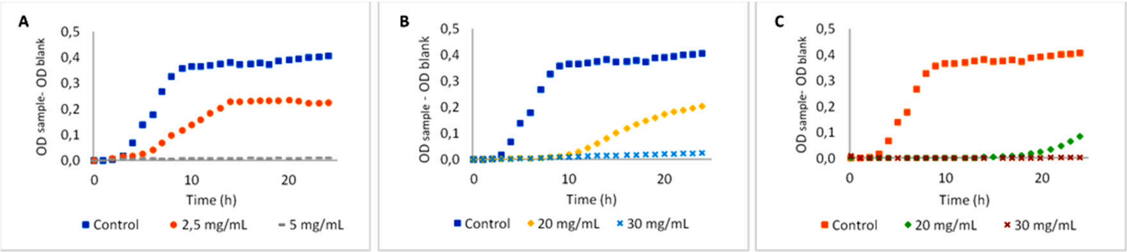

(a)

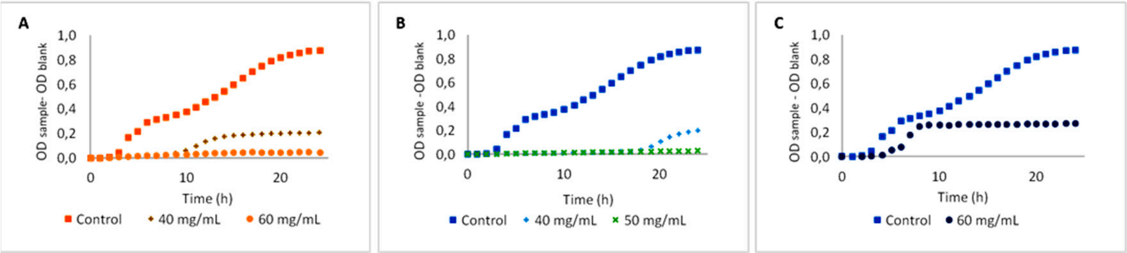

(b)

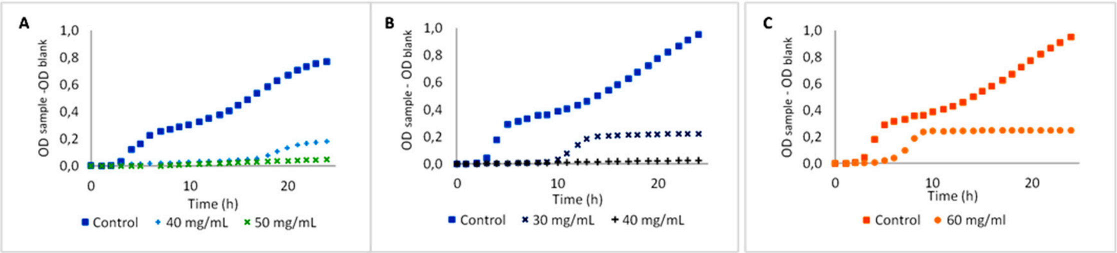

(c)

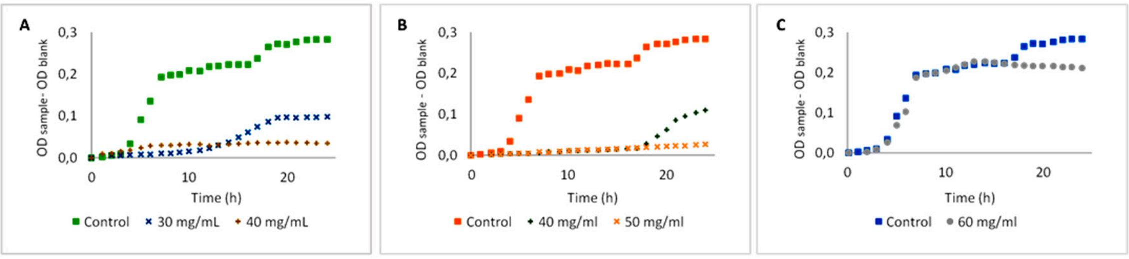

(d)

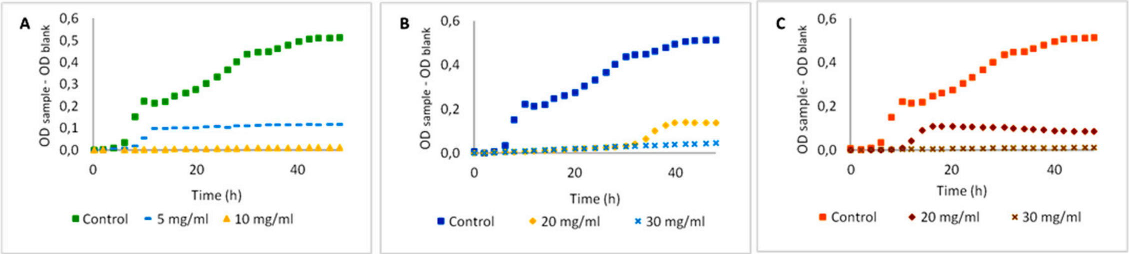

(e)

Figure S1. Growth of (a) *S. aureus*, (b) *S. Typhimurium*, (c) *E. coli*, (d) *L. monocytogenes* and (e) *Y. enterocolitica* in broth with olive leaf extracts added, where A, B and C are the MAE-W, MAE-Et50 and MAE-Gly5 extracts, respectively.

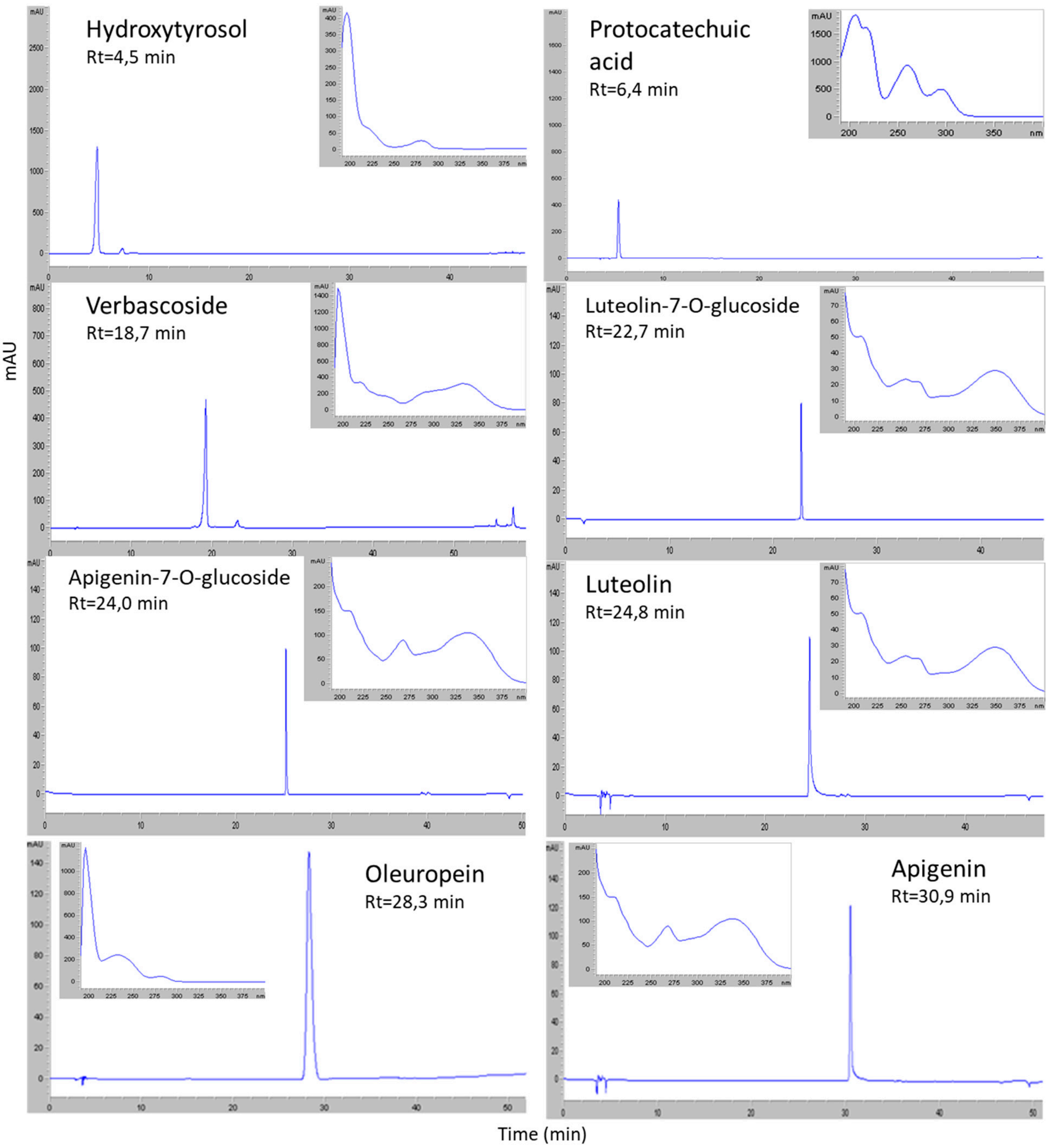

Figure S2. HPLC-UV chromatograms at 280 nm, the UV spectra and the retention time (Rt) of the phenolic standards employed to investigate and quantify these compounds in the phenolic extracts.

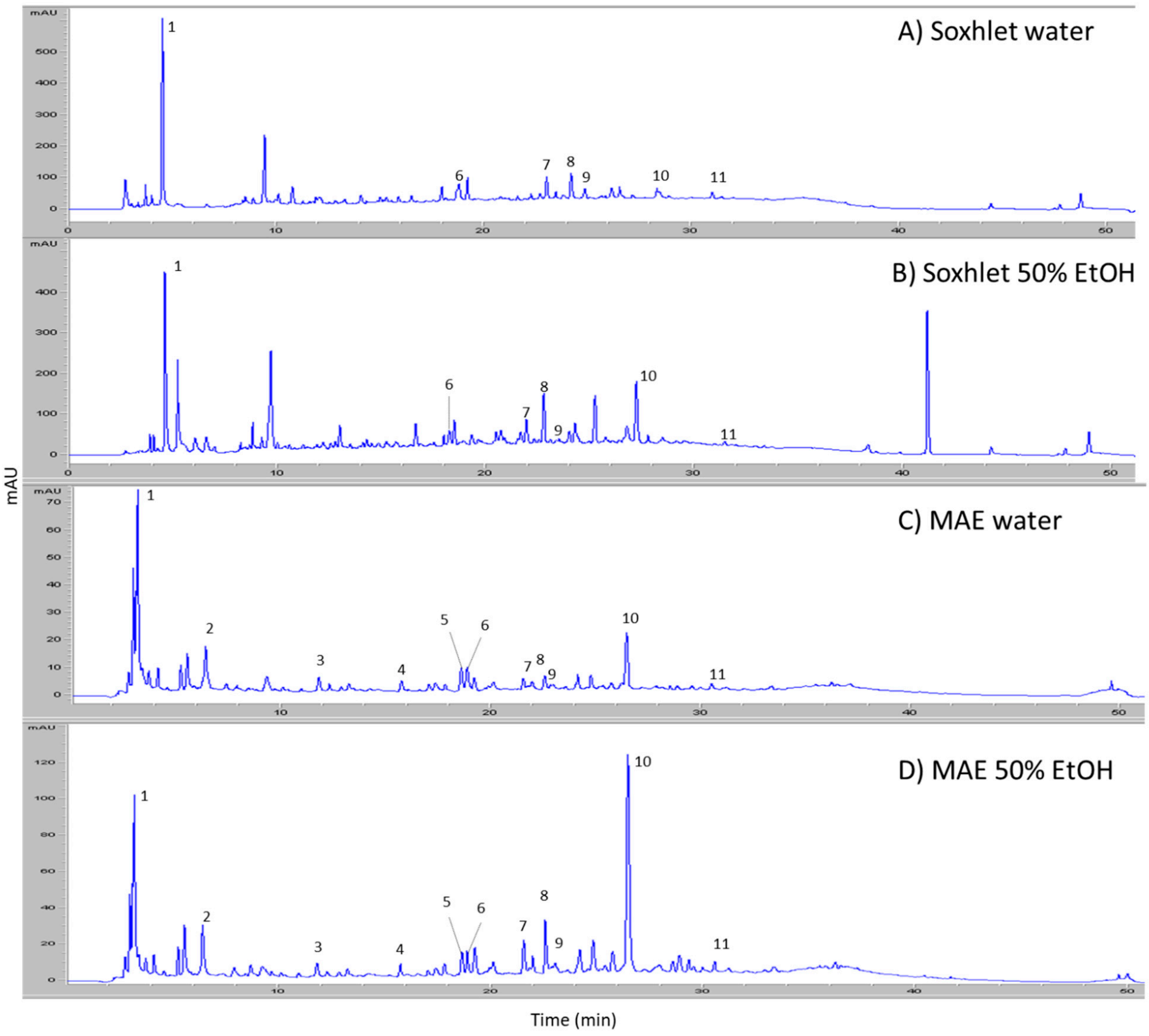

Figure S3. HPLC-UV chromatograms at 280 nm of the phenolic extracts obtained by two methods and two solvents: (A) Soxhlet water; (B) Soxhlet 50% EtOH; (C) Microwave-Assisted Extraction at 80°C during 10 min (MAE 10-80) water; (D) MAE 10-80 50% EtOH. Quantified compounds were: (1) hydroxytyrosol, (2) protocatechuic acid, (3) elenolic acid derivative-1, (4) elenolic acid derivative-2, (5) elenolic acid derivative-3, (6) verbascoside, (7) luteolin-7-O-glucoside, (8) apigenin-7-O-glucoside, (9) luteolin, (10) oleuropein, and (11) apigenin.
